# Supplementary material for: Loss of Asxl2 leads to myeloid malignancies in mice
Source: Nat Commun. 2017 Jun 8;8:15456. doi: 10.1038/ncomms15456 (PMC5472177; doi:10.1038/ncomms15456)
Supplement: Supplementary Information — Supplementary Figures and Supplementary Tables [file ncomms15456-s1.pdf]

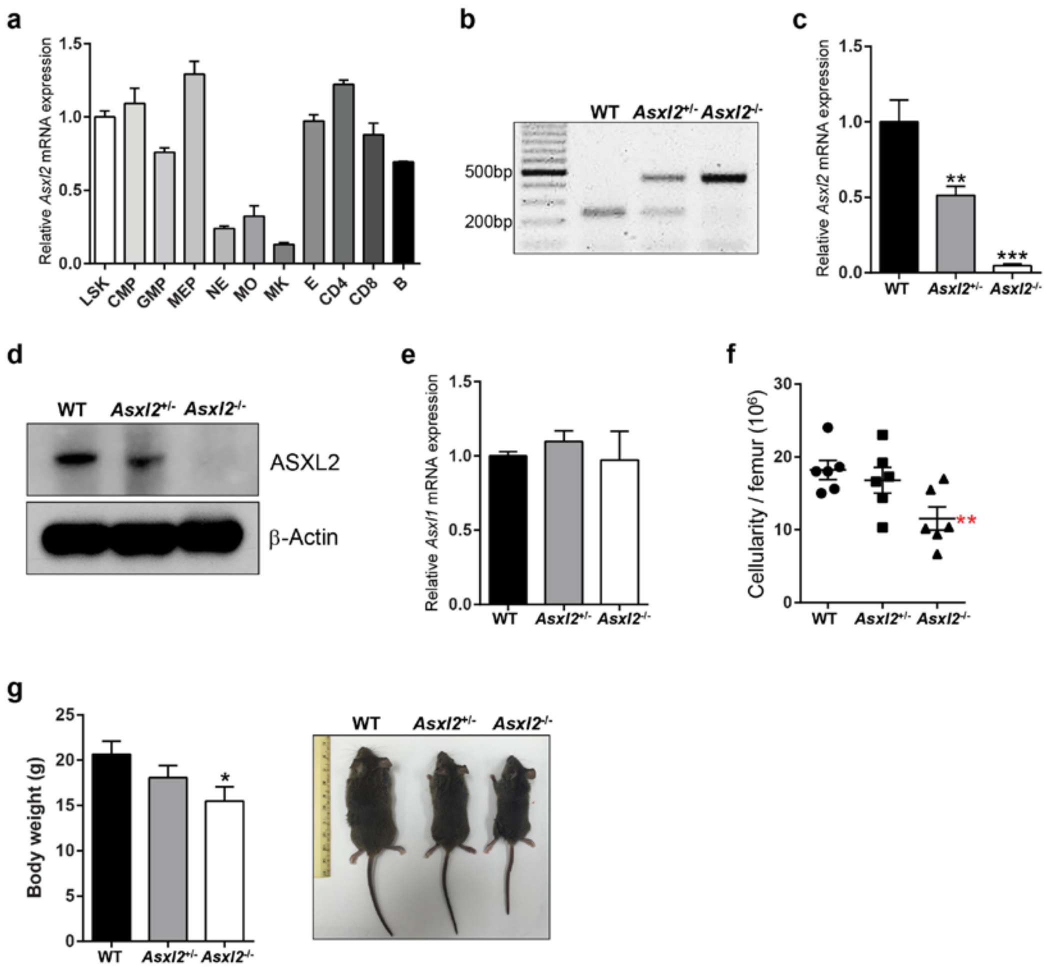

Supplementary Fig. 1: Characterization of *Asxl2*$^{-/-}$ mouse model. (a) HSCs and their differentiated cell populations were sorted from the BM of WT mice using respective surface markers, and *Asxl2* mRNA expression was detected by qPCR (normalized to *Actb*). PCR results were from 3 independent experiments. LSK, Lin^{-}Sca1^{+}cKit^{+} cells; CMP, common myeloid progenitor cells; GMP, granulocyte-monocyte progenitor cells; MEP, megakaryocyte-erythrocyte progenitor cells; NE, neutrophils; MO, monocytes; E, erythrocytes; MK, megakaryocytes. **(b)** The genotypes of WT, *Asxl2*$^{+/-}$ and *Asxl2*$^{-/-}$ mice were verified by PCR. The WT allele produces a band of 250 bp, while the *Asxl2*-mutant allele produces a band of 480 bp. **(c)** *Asxl2* mRNA expression was detected in the BMMNCs from WT, *Asxl2*$^{+/-}$ and *Asxl2*$^{-/-}$ mice (6-8 week old, 3 mice/genotype) by qPCR

with three replicates. **(d)** ASXL2 protein levels were detected in the BMMNCs from WT, *Asxl2*$^{+/-}$ and *Asxl2*$^{-/-}$ mice (6-week old) by Western blot. **(e)** *Asxl1* mRNA expression was detected in the BMMNCs from WT, *Asxl2*$^{+/-}$ and *Asxl2*$^{-/-}$ mice (6-8 week old, 3 mice/genotype) with three replicates. **(f)** Bone marrow cellularity (femur) for each genotype of mice (n=6 mice/genotype). **(g)** Body weight of WT, *Asxl2*$^{+/-}$ and *Asxl2*$^{-/-}$ mice (6-8 week old, 6 mice/genotype). The gross appearance of representative mice from each genotype (6-week old). Data are presented as mean ± s.e.m. Comparisons among groups were performed by one-way ANOVA. $^*P < 0.05$, $^{**}P < 0.01$, $^{***}P < 0.001$.

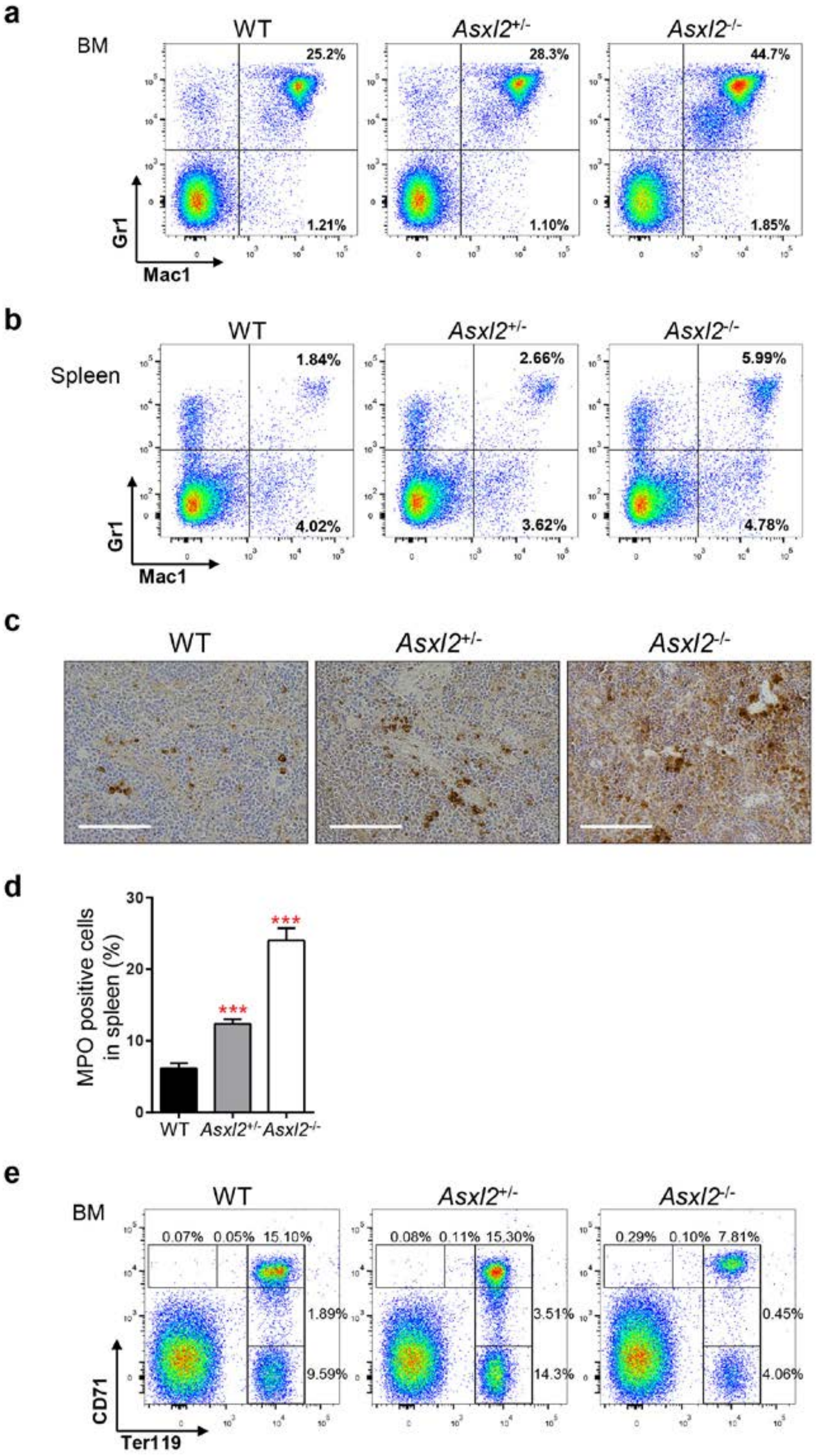

Supplementary Fig. 2: Loss of *Asxl2* alters hematopoiesis *in vivo*. (a) Flow cytometric analysis of Gr1^{+}/Mac1^{+} cell populations in BM of representative WT, *Asxl2*$^{+/-}$, and *Asxl2*$^{-/-}$ mice (6-week old). **(b)** Flow cytometric analysis of Gr1^{+}/Mac1^{+} cell

populations in spleens of representative WT, *Asxl2*$^{+/-}$, and *Asxl2*$^{-/-}$ mice (6-week old). **(c)** Myeloperoxidase (MPO) staining of paraffin-embedded sections of spleens from representative WT, *Asxl2*$^{+/-}$ and *Asxl2*$^{-/-}$ mice (6-week old). The MPO-positive cells were shown as brown particles in the cytoplasm. Scale bar = 100 μm. **(d)** Quantitation of the percent MPO-positive cells in the spleens of WT, *Asxl2*$^{+/-}$ and *Asxl2*$^{-/-}$ mice (6-8 week old, 3 mice/genotype). (**e**) Flow cytometric analysis of CD71^{+}/Ter119^{+} cell populations in BM of representative WT, *Asxl2*$^{+/-}$, and *Asxl2*$^{-/-}$ mice (6-week old). Data are presented as mean ± s.e.m. Comparisons between two groups were performed by one-way ANOVA. ***P < 0.001.

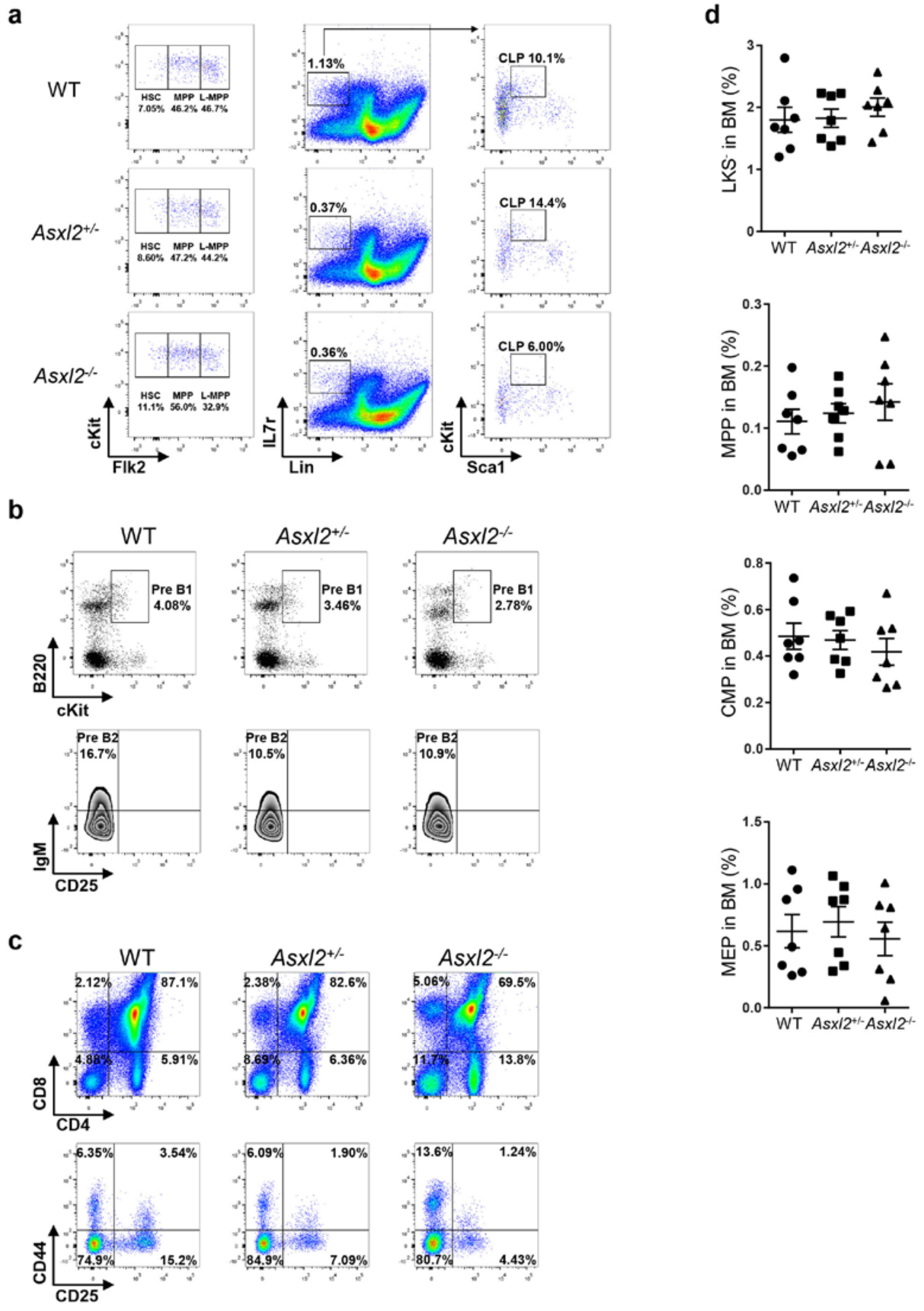

Supplementary Fig. 3: Loss of *Asxl2* impairs the development of lymphocytes. (a) Flow cytometric analysis of LMPP and CLP cell populations in BM of representative WT, *Asxl2*$^{+/-}$ and *Asxl2*$^{-/-}$ mice (6-week old). **(b)** Flow cytometric analysis of B cell subpopulations in BM of representative WT, *Asxl2*$^{+/-}$ and *Asxl2*$^{-/-}$ mice (6-week old). **(c)**

Flow cytometric analysis of T cell subpopulations in thymus of representative WT, *Asxl2*$^{+/-}$ and *Asxl2*$^{-/-}$ mice (6-week old). **(d)** Flow cytometric analysis of percent LKS^{-}, MPP, CMP, and MEP in total BM cells of WT, *Asxl2*$^{+/-}$ and *Asxl2*$^{-/-}$ mice (6-8 week old, 7 mice/genotype). Data are presented as mean ± s.e.m. Comparisons between two groups were performed by one-way ANOVA.

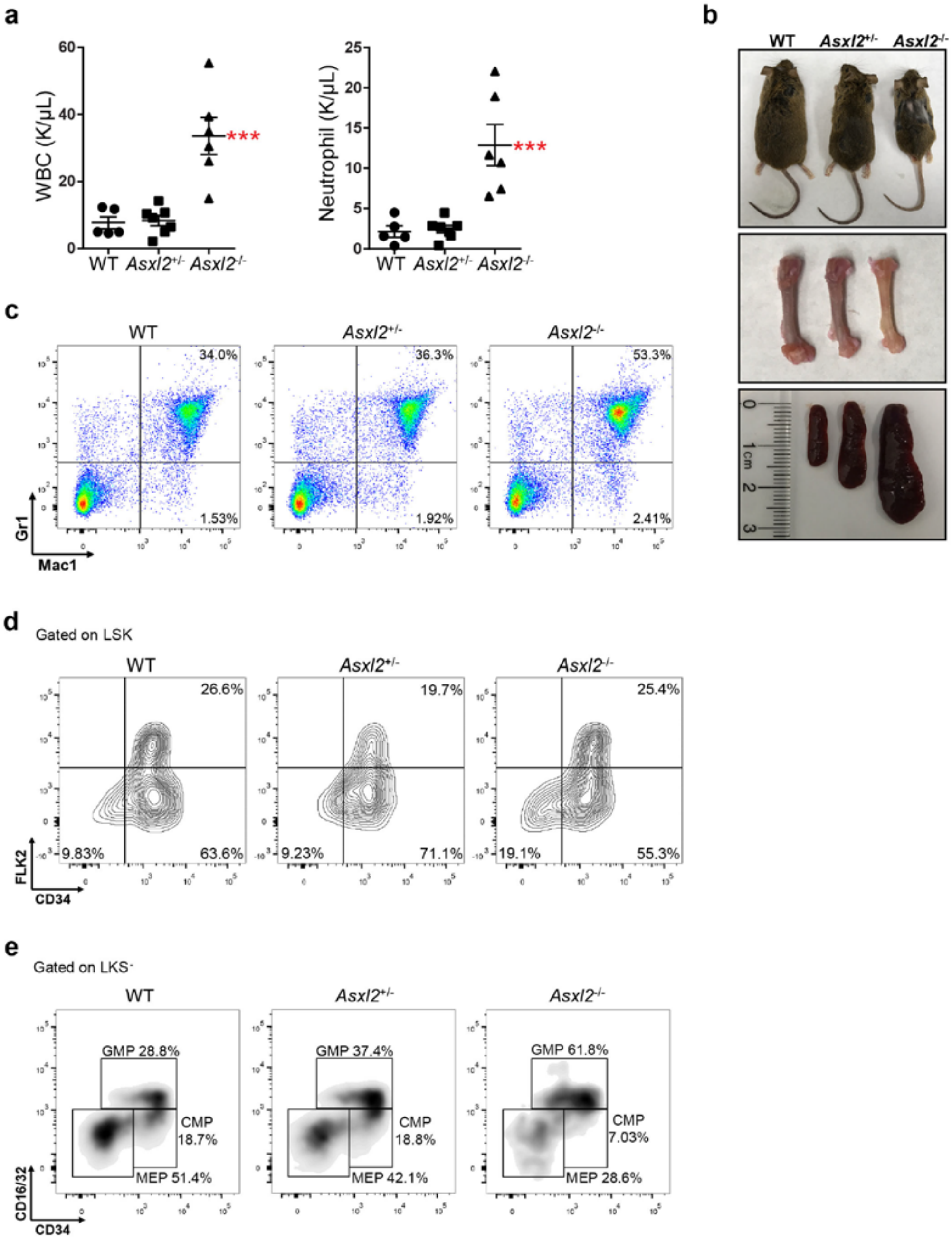

Supplementary Fig. 4: Loss of *Asxl2* alters hematopoiesis in aged mice. (a) WBC and neutrophil counts of PB from aged WT (n=5), *Asxl2*$^{+/-}$ (n=7), and *Asxl2*$^{-/-}$ (n=6) mice at 12 months of age were shown. **(b)** Gross appearance of representative body, femurs and spleens from each genotype of mice (12-month old). **(c)** Flow cytometric analysis of Gr1^{+}/Mac1^{+} cell populations in PB of representative aged WT, *Asxl2*$^{+/-}$, and *Asxl2*$^{-/-}$ mice

(12-month old). **(d)** Flow cytometric analysis of LT-HSC, ST-HSC and MPP compartments in BM LSK cells of representative aged WT, *Asxl2*$^{+/-}$, and *Asxl2*$^{-/-}$ mice (12-month old). **(e)** Flow cytometric analysis of CMP, GMP, and MEP populations in BM LKS^{-} cells of representative aged WT, *Asxl2*$^{+/-}$, and *Asxl2*$^{-/-}$ mice (12-month old). Data are presented as mean ± s.e.m. Comparisons between two groups were performed by one-way ANOVA. ***$P < 0.001$.

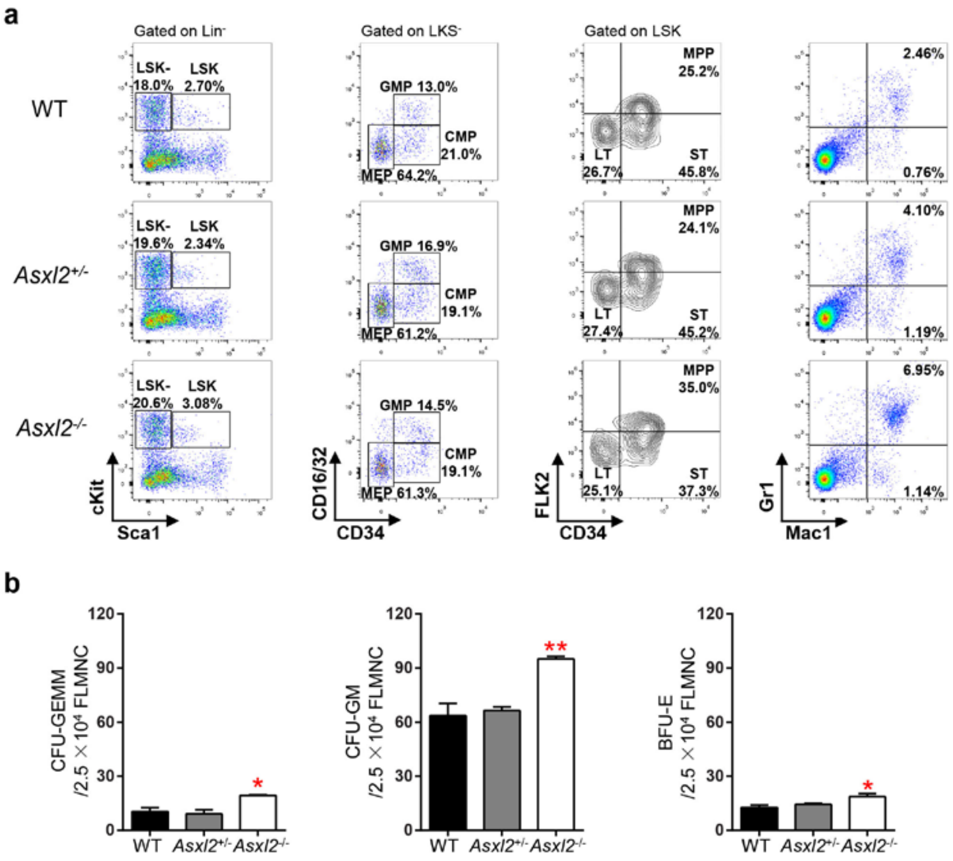

Supplementary Fig. 5: Loss of *Asxl2* alters hematopoiesis in fetal liver. (a) Flow cytometric analysis of HSC/HPC and Gr1^{+}/Mac1^{+} cell populations in fetal livers from WT, *Asxl2*$^{+/-}$ and *Asxl2*$^{-/-}$ embryos (E12.5). **(b)** Colony-forming units (CFU-C) in fetal livers from WT, *Asxl2*$^{+/-}$ and *Asxl2*$^{-/-}$ embryos (E12.5) were assessed in semi-solid media in the presence of mSCF, mIL-3, hIL-6, mTPO, mGM-CSF, and mEPO. The colony assays were performed in three replicates. Data are presented as mean ± s.e.m. Comparisons between two groups were performed by one-way ANOVA. *$P < 0.05$, **$P < 0.01$.

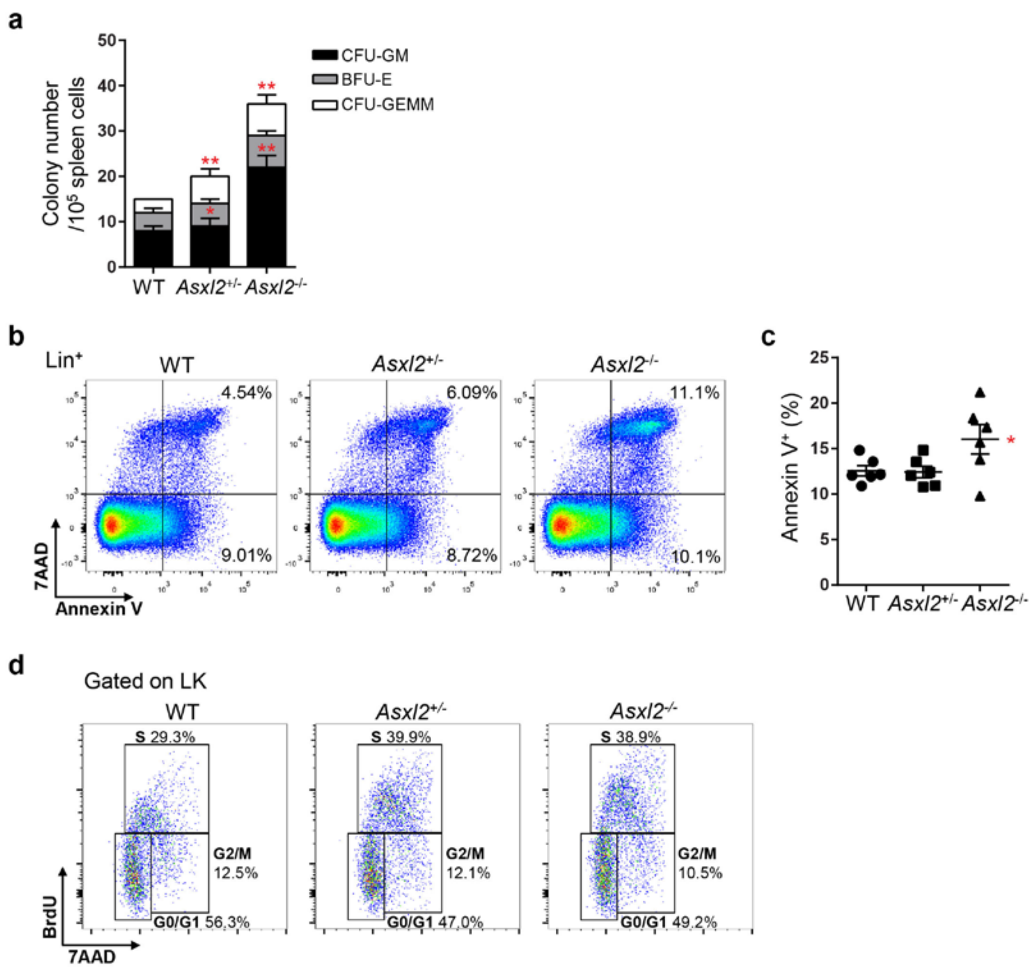

Supplementary Fig. 6: Loss of *Asxl2* increases CFU-Cs in spleen and alters apoptosis and cell cycle of hematopoietic cells. (a) CFU numbers of spleen cells from 3 sets of WT, *Asxl2*$^{+/-}$ and *Asxl2*$^{-/-}$ mice were assessed in semi-solid media in the presence of mSCF, mIL-3, hIL-6, mTPO, mGM-CSF, and mEPO. The colony assays were performed in three replicates. **(b)** Flow cytometric analysis (Annexin V/7-AAD staining) of freshly isolated Lin^{+} cells from BM of representative WT, *Asxl2*$^{+/-}$, and *Asxl2*$^{-/-}$ mice are shown. **(c)** Quantitation of the Annexin V^{+} apoptotic cells in BM Lin^{+} cells of WT, *Asxl2*$^{+/-}$, and *Asxl2*$^{-/-}$ mice (6-8 week old, 6 mice/genotype). **(d)** Flow cytometric analysis of cell cycle of LK cells in BM of representative WT, *Asxl2*$^{+/-}$ and *Asxl2*$^{-/-}$ mice. Data are

presented as mean ± s.e.m. Comparisons between two groups were performed by one-way ANOVA. *$P < 0.05$, **$P < 0.01$.

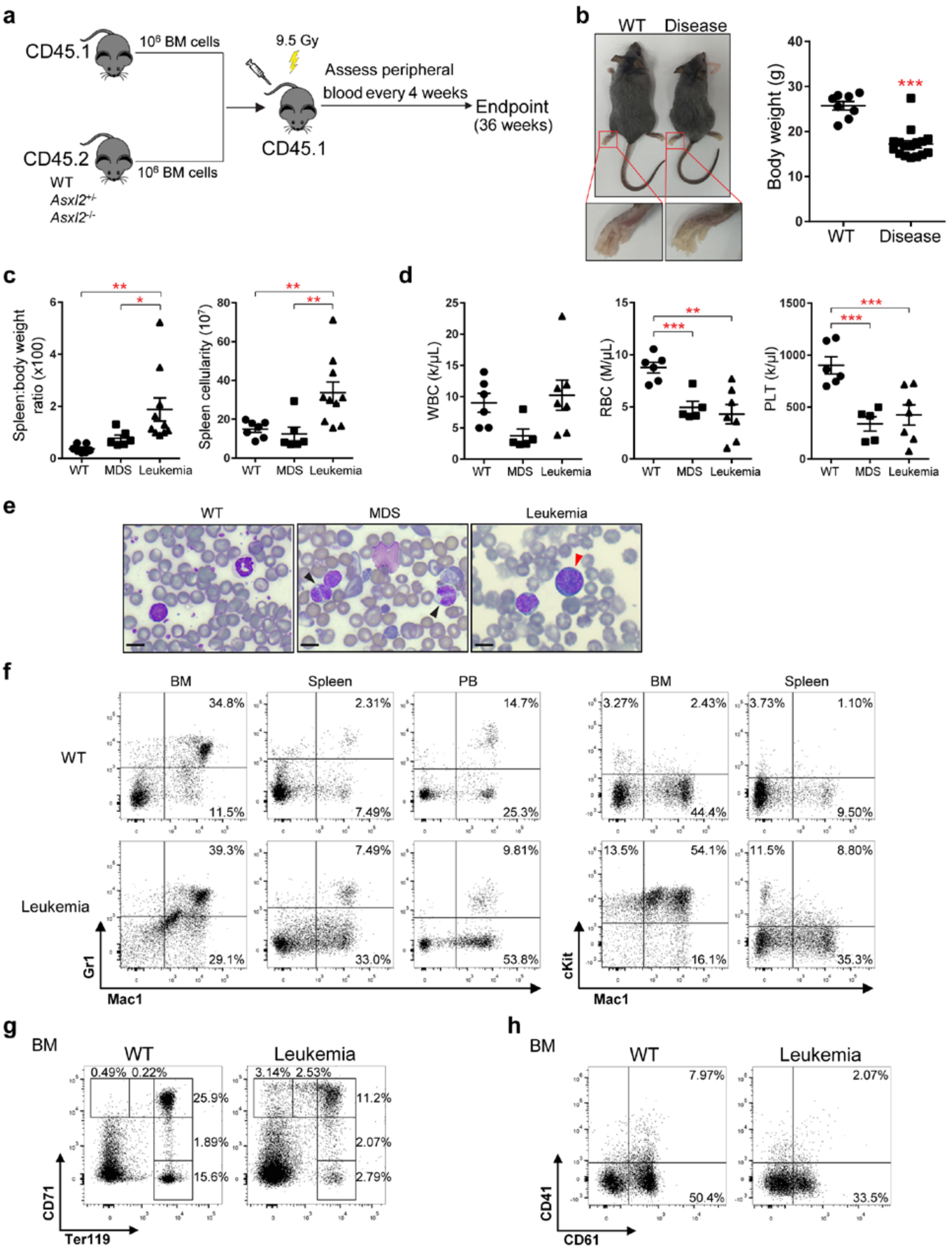
a
CD45.1
10^6 BM cells
CD45.2
WT
Asxl2+/-
Asxl2-/-
10^6 BM cells
9.5 Gy
CD45.1
Assess peripheral blood every 4 weeks
Endpoint (36 weeks)
b
WT
Disease
Body weight (g)
***
c
Spleen:body weight ratio (x100)
Spleen cellularity (10^7)
WT
MDS
Leukemia
d
WBC (k/μL)
RBC (M/μL)
PLT (k/μl)
e
WT
MDS
Leukemia
f
BM
Spleen
PB
WT
Leukemia
Gr1
Mac1
cKit
34.8%
11.5%
2.31%
7.49%
14.7%
25.3%
3.27%
2.43%
44.4%
3.73%
1.10%
9.50%
39.3%
29.1%
7.49%
33.0%
9.81%
53.8%
13.5%
54.1%
16.1%
11.5%
8.80%
35.3%
g
BM
CD71
Ter119
0.49%
0.22%
25.9%
1.89%
15.6%
3.14%
2.53%
11.2%
2.07%
2.79%
h
CD41
CD61
7.97%
50.4%
2.07%
33.5%

Supplementary Fig. 7: Cell-autonomous effect of myeloid leukemia transformation in *Asxl2*$^{+/-}$ and *Asxl2*$^{-/-}$ hematopoietic cells. (a) Schematic diagram of competitive BM transplantation. CD45.2^{+} BM cells from WT, *Asxl2*$^{+/-}$ or *Asxl2*$^{-/-}$ mice were mixed with CD45.1^{+} competitor cells at a ratio of 1:1 (1x10^{6} cells each) and transplanted into lethally irradiated recipients. **(b)** Gross appearance, feet pads and body weight of recipients receiving WT BM cells (n=8 mice), *Asxl2*$^{+/-}$ or *Asxl2*$^{-/-}$ BM cells (n=16 mice). **(c)** Spleen/body weight ratio (×100) and spleen cellularity of recipient mice (WT recipients: n=7; *Asxl2*$^{+/-}$ or *Asxl2*$^{-/-}$ recipients, MDS: n=6, leukemia: n=10). **(d)** Parameters of PB from each transplantation group were summarized. WT recipients: n=6; *Asxl2*$^{+/-}$ or *Asxl2*$^{-/-}$ recipients, MDS: n=5, leukemia: n=7. **(e)** Representative images of May-Grünwald-Giemsa–stained PB smear preparations from representative MDS and leukemic recipients transplanted with *Asxl2*$^{-/-}$ BM cells. Scale bar = 10 μm. Red arrow indicate blast cells and black arrows indicate dysplastic cells. **(f-h)** Flow cytometric analysis of myeloid cell (Gr1^{+}/Mac1^{+}), myeloblast (cKit^{+}/Mac1^{+}), erythroid (CD71^{+}/Ter119^{+}) and megakaryocyte (CD61^{+}/CD41^{+}) populations in the BM, and/or spleen/PB of representative recipient mice. Data are presented as mean ± s.e.m. Comparisons between two groups were performed by two-tailed unpaired Student's *t*-test. Comparisons among groups were performed by one-way ANOVA. *$P < 0.05$, **$P < 0.01$, ***$P < 0.001$.

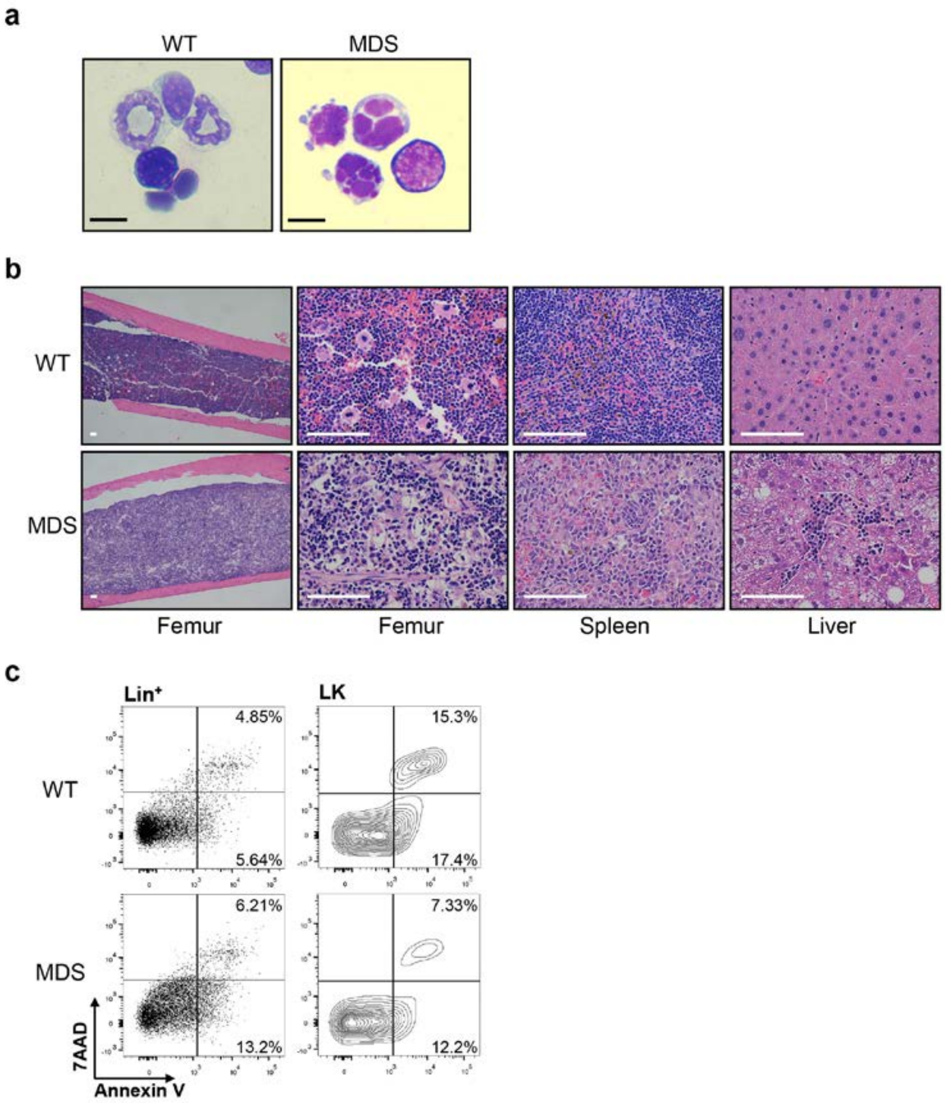

Supplementary Fig. 8: Development of MDS-like disease in recipients transplanted with BM cells from *Asxl2*$^{-/-}$ mice. (a,b) Representative images of May-Grünwald-Giemsa–stained cytospin preparations **(a)** and H&E stained histological sections of femur, spleen, and liver **(b)** from representative MDS recipients transplanted with *Asxl2*$^{-/-}$ BM cells and a recipient transplanted with WT BM cells. Scale bar = 100 μm. **(c)** Apoptosis analysis (Annexin V/7-AAD staining) of Lin$^+$ and LK cells from BM of representative MDS recipients transplanted with *Asxl2*$^{-/-}$ BM cells and a control recipient transplanted with WT BM cells are shown.

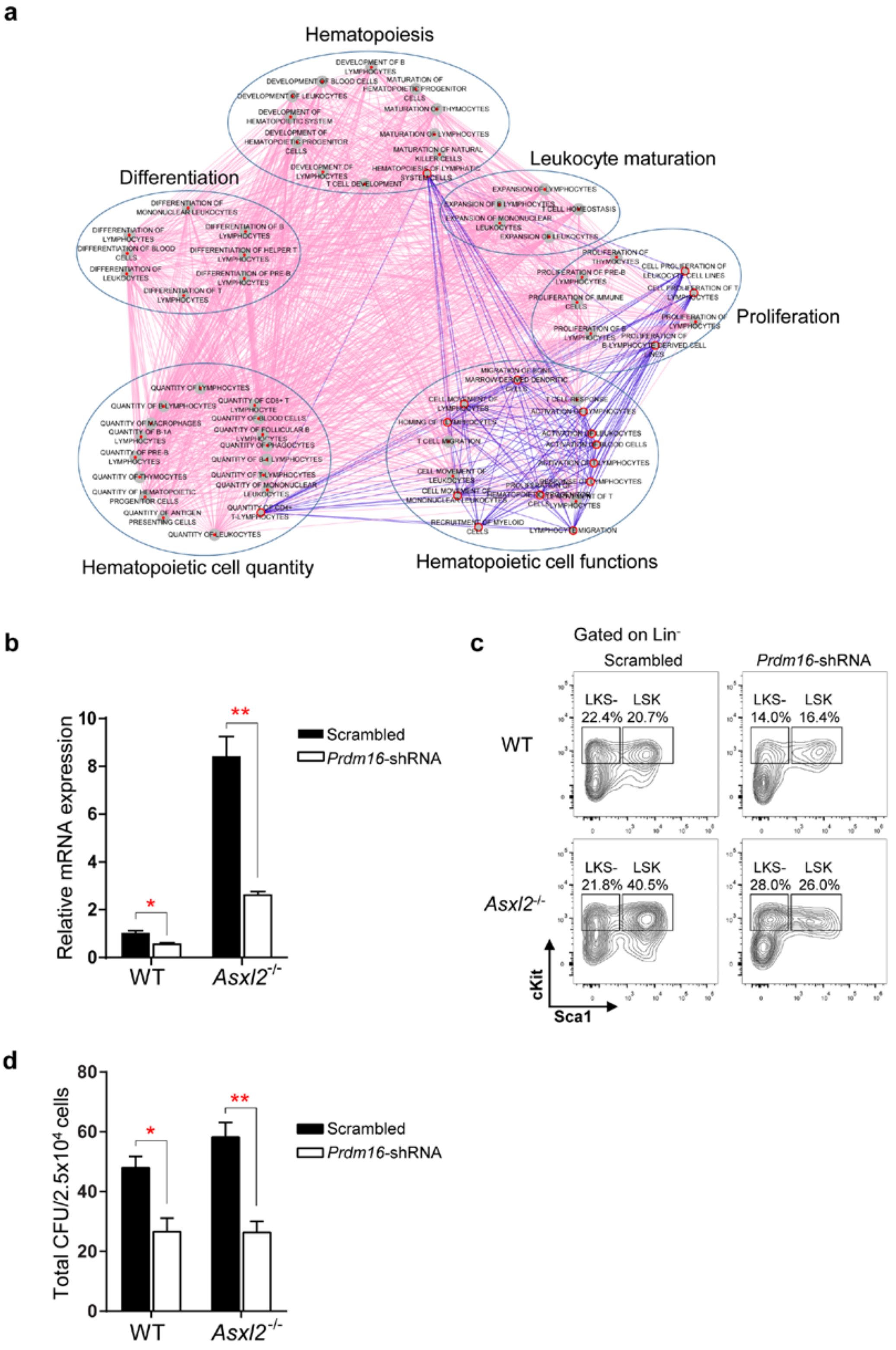
a
Hematopoiesis
Differentiation
Leukocyte maturation
Proliferation
Hematopoietic cell quantity
Hematopoietic cell functions
b
Relative mRNA expression
Scrambled
Prdm16-shRNA
WT
Asxl2-/-
c
Gated on Lin-
Scrambled
Prdm16-shRNA
WT
LKS- LSK
22.4% 20.7%
14.0% 16.4%
Asxl2-/-
21.8% 40.5%
28.0% 26.0%
cKit
Sca1
d
Total CFU/2.5x10^4 cells
Scrambled
Prdm16-shRNA
WT
Asxl2-/-

Supplementary Fig. 9: *Asxl2* deletion alters the expression of genes associated with hematopoiesis in LK cells. (a) Enrichment map was used for visualizing the network of hematological system-related Diseases and Biological Functions enriched with 565 DEGs ($P < 0.05$) using IPA. Edges indicate gene overlap between the enriched terms; thickness represents statistical significance of term enrichment. Only edges with a Fisher's Exact Test nominal $P < 10^{-4}$ were visualized. Terms linked by red lines with positive z-scores indicate that functional activity is increased, whereas that by blue ones with negative z-scores mean decreased activity. Oval indicates functional cluster, which includes relevant terms. **(b)** qPCR showing the mRNA expression of *Prdm16* after *Prdm16*-knockdown (KD) (n=3) with three replicates. **(c)** Flow cytometric analysis of Lin^- cells from WT and $Asxl2^{-/-}$ fetal liver after *Prdm16*-KD for seven days. **(d)** CFU-C in WT and $Asxl2^{-/-}$ fetal liver cells after *Prdm16*-KD for seven days (n=3). The colony assays were performed in three replicates. Data are presented as mean ± s.e.m. Comparisons between two groups were performed by two-tailed paired Student's *t*-test.$^*P < 0.05$, $^{**}P < 0.01$.

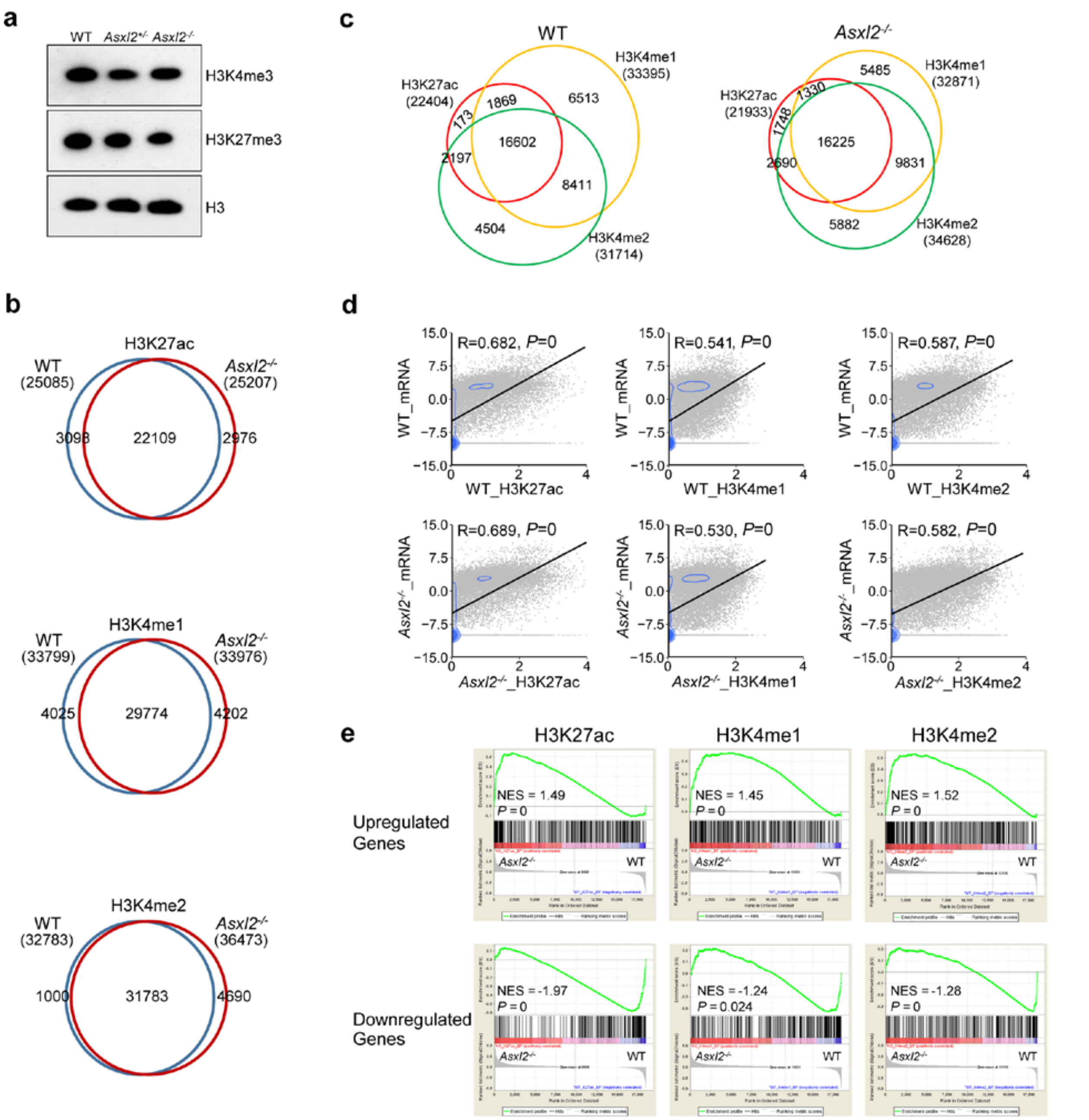

Supplementary Fig. 10: ASXL2 regulates gene expression through histone modifications. **(a)** Western blot analysis of Histone H3 modifications in WT and *Asxl2*$^{-/-}$ LK cells. **(b,c)** Venn diagram shows the overlap among enriched regions in H3K27ac, H3K4me1 and H3K4me2 between WT and *Asxl2*$^{-/-}$ LK cells, respectively. **(d)** Scatter plots with kernel density estimation showing correlations between histone modifications and

gene expression for all genes in WT and *Asxl2*$^{-/-}$ LK cells. X-axis shows enrichment fold of histone modification. Y-axis shows the $\log_2$ transformed expression level. Solid line, linear fit. Spearman correlation coefficient *R* and *P* values are shown. **(e)** GSEA plots showing changes in histone modifications between WT and *Asxl2*$^{-/-}$ LK cells for upregulated and downregulated genes. The normalized enrichment score (NES) and *P* value are shown**.**

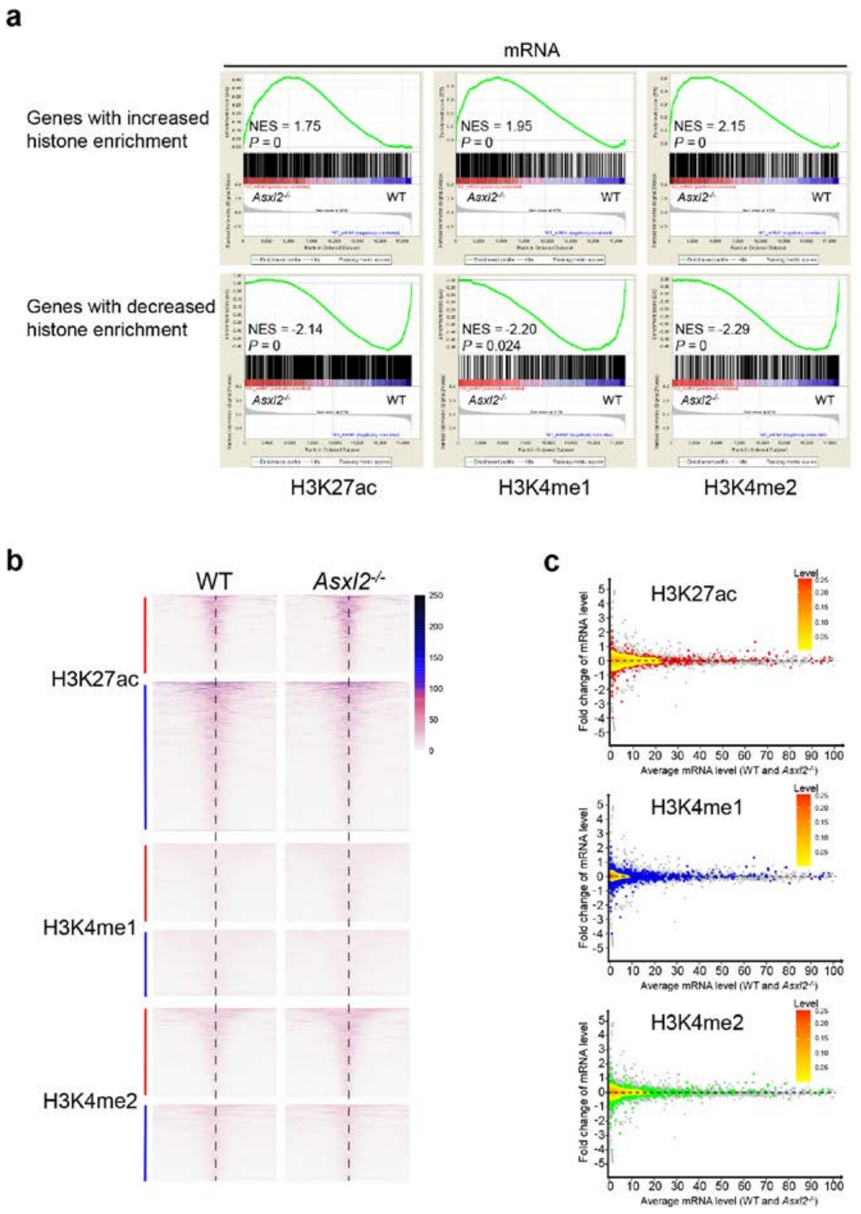

Supplementary Fig. 11: ASXL2 regulates gene expression through H3K27ac and H3K4me1/2. **(a)** GSEA plots showing changes in mRNA expression between WT and *Asxl2*$^{-/-}$ LK cells for genes with increased and decreased histone signals. NES and *P* are shown. **(b)** The heatmap shows the ChIP-seq signals in H3K27ac, H3K4me1 and H3K4me2 at 5 Kb upstream and downstream regions across the centers of DERs in WT and *Asxl2*$^{-/-}$ LK cells, respectively. Color scale indicates ChIP-seq signal in reads per

kilobase per million reads (RPKM). Red and blue bars indicate DERs with increased and decreased signals in each histone mark in *Asxl2*$^{-/-}$ cells compared to WT cells, respectively. **(c)** The 2D Kernel Density plots displaying the change and level of mRNA expression for all expressed genes. X- and Y-axes represent the average level and $\log_2$FC of mRNA expression between WT and *Asxl2*$^{-/-}$, respectively. Grey, red, green and blue spots indicate all expressed genes and genes with DERs in H3K27ac, H3K4me1, and H3K4me2, respectively. The color scale shows the density of the data at 5, 10, 15, 20 and 25 percentiles.

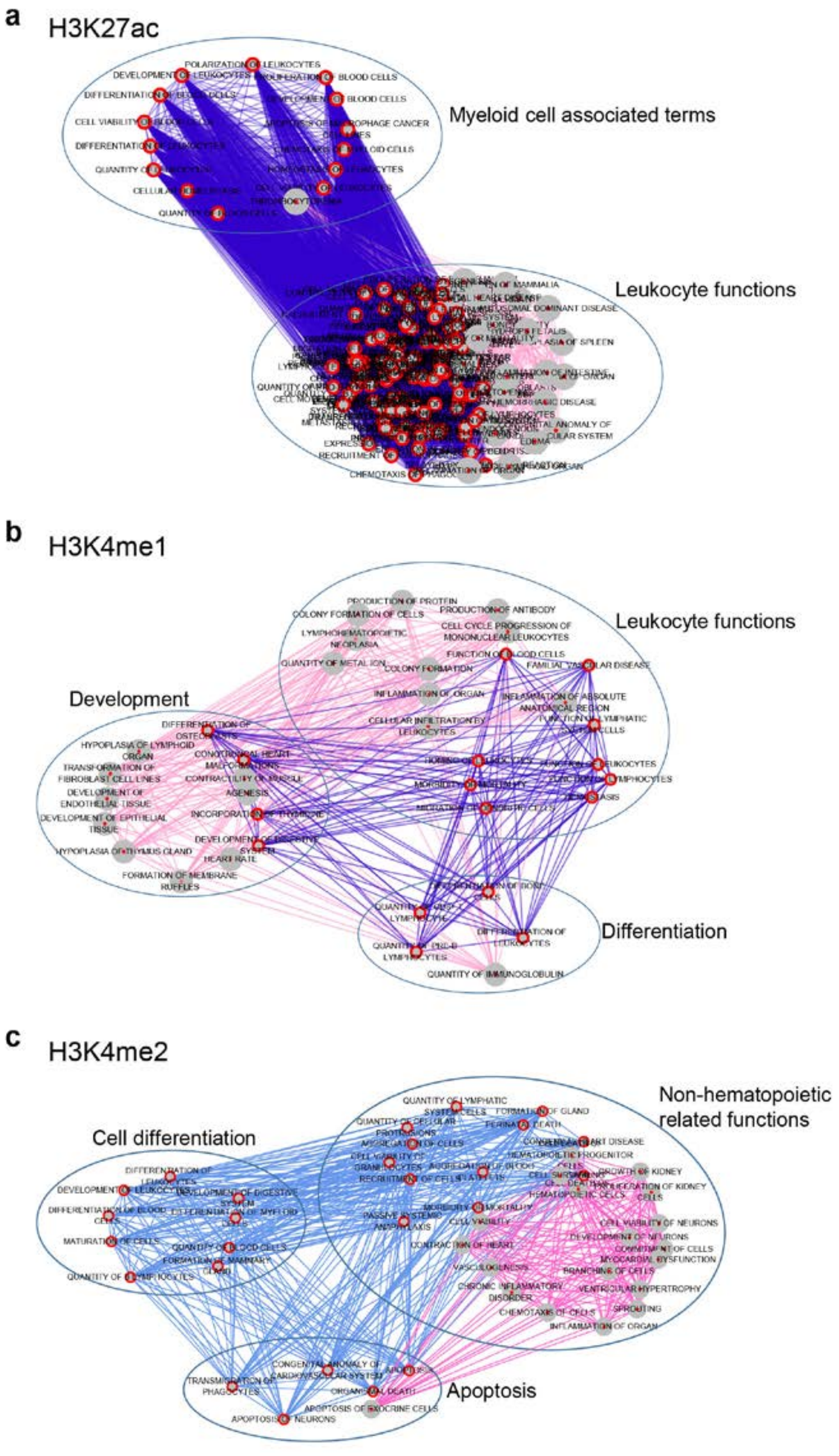

Supplementary Fig. 12: Genes with the differential signals in H3K27ac are enriched in myeloid and hematopoietic signatures. (a-c) Enrichment map was used for visualizing the network of diseases and biological functions enriched with genes using

IPA, which were identified to have differential enrichment signal in H3K27ac **(a),** H3K4me1 **(b)** and H3K4me2 **(c)** between WT and *Asxl2*$^{-/-}$ LK cells. The network of enriched functional terms was shown (Nodes, P < 0.01 and |z-score| ≥ 2, 1.5 and 1.5 for H3K27ac, H3K4me1 and H3K4me2 in IPA respectively). Edges indicate gene overlap between the enriched terms; thickness represents statistical significance of term enrichment. Only edges with a Fisher's Exact Test nominal $P < 10^{-4}$ were visualized. Terms linked by red lines with positive z-scores indicate that functional activity is increased, whereas that by blue ones with negative z-scores mean decreased activity. Oval indicates functional cluster, which includes relevant terms.

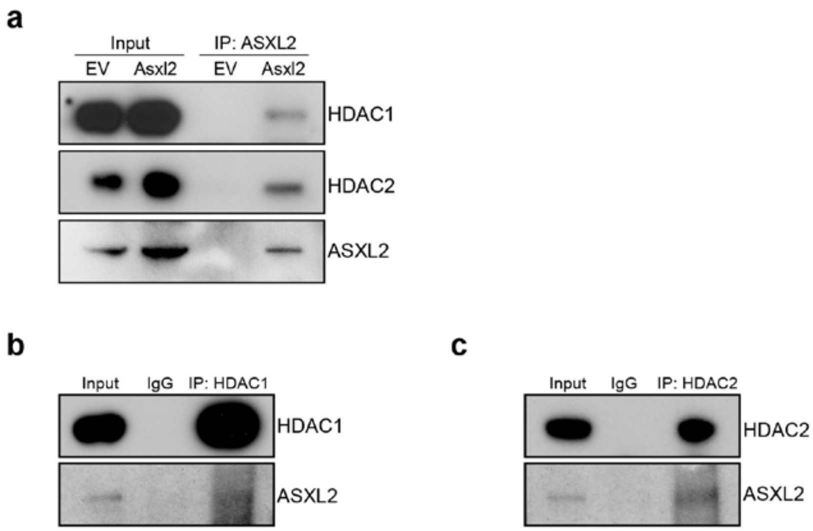

Supplementary Fig. 13: ASXL2 associates with HDAC1 and HDAC2 in 293T cells by IP. (a) Nuclear fractions were extracted from 293T cells (ATCC) transfected with pCDNA3.1^{+} (empty vector, EV) and FLAG-tagged ASXL2 (ASXL2) and subjected to Immunoprecipitation (IP) using anti-ASXL2 antibody. Western blots showed the association of ASXL2 with HDAC1 and HDAC2 by IP. **(b,c)** Reciprocal IP and Western blots showed the endogenous interaction between ASXL2 and HDAC1 (b) or HDAC2 (c).

Supplementary Figure 1d

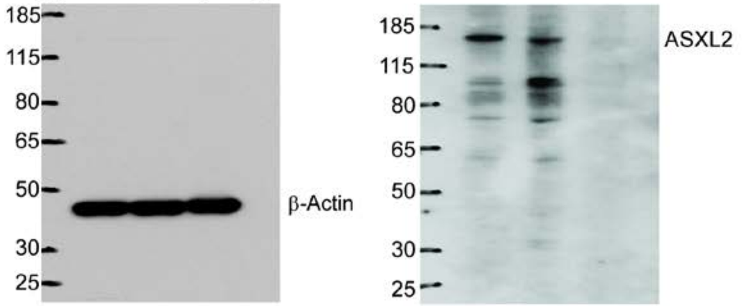

Supplementary Figure 10a

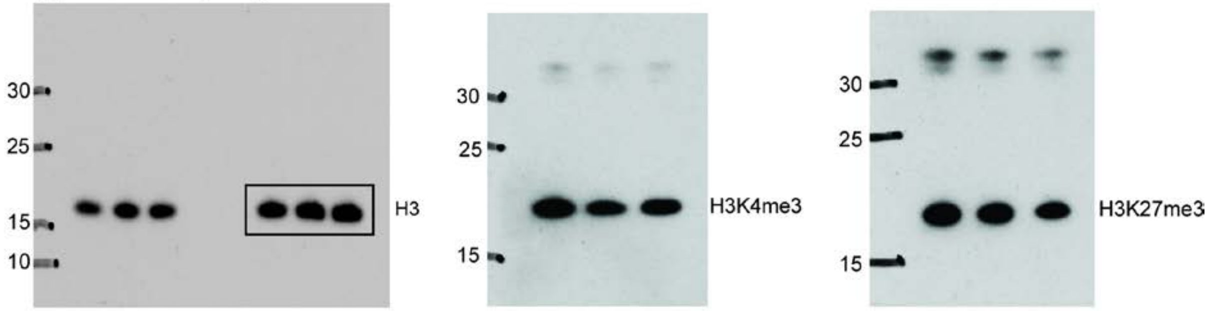

Supplementary Figure 13a

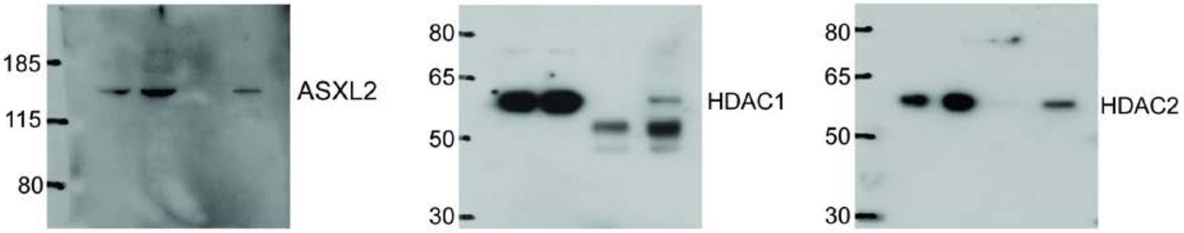

Supplementary Figure 13b and 13c

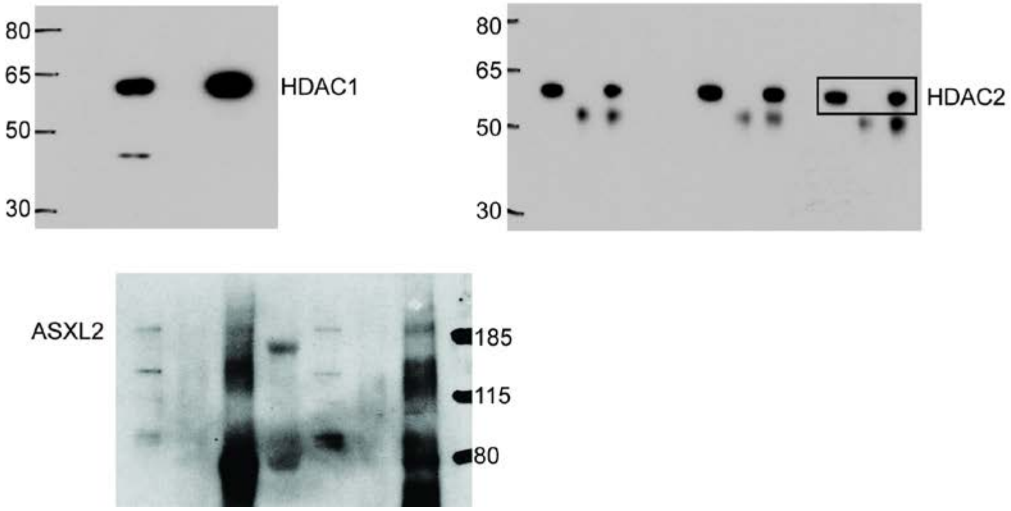

Supplementary Figure 14. Scan images of gel electrophoresis.

Supplementary Table 1. Antibodies used for flow cytometry.

Antibody name	Clone	Color	Catalog	Company
Rat anti-Mouse CD34	RAM34	FITC	553733	BD Pharmingen
Anti-mouse CD34	RAM34	Alexa Fluor 700	56-0341-82	eBioscience
Rat anti-Mouse Ly-6A/E (Sca1)	D7	PE-Cy7	558162	BD Pharmingen
Rat anti-Mouse CD117	2B8	PE	553355	BD Pharmingen
Rat anti-Mouse CD117	2B8	APC	553356	BD Pharmingen
Rat anti-Mouse CD117	2B8	PerCP-Cy5.5	560557	BD Pharmingen
Rat anti-mouse CD117	2B8	FITC	553354	BD Pharmingen
Rat anti-Mouse CD16/CD32	2.4G2	APC-Cy7	560541	BD Pharmingen
Rat anti-mouse CD135	A2F10.1	BV421	562898	BD Horizon
Mouse Lineage Antibody Cocktail		APC	51-9003632	BD Pharmingen
Rat anti-Mouse CD71	C2	FITC	553266	BD Pharmingen
Rat anti-Mouse CD71	C2	PE	553267	BD Pharmingen
Rat anti-Mouse TER-119	TER-119	APC	557909	BD Pharmingen
Anti-Mouse CD41	MWReg30	APC	17-0411-80	eBioscience
Anti-Mouse/Rat CD61	2C9.G3	PE	12-0611-81	eBioscience
Rat anti-Mouse Ly-6G and Ly-6C	RB6-8c5	PerCP-Cy5.5	552093	BD Pharmingen
Rat anti-Mouse Ly-6G and Ly-6C	RB6-8c5	PE-Cy7	552985	BD Pharmingen
Rat anti-Mouse CD11b	M1/70	PE	553311	BD Pharmingen
Rat anti-Mouse IgM	II/41	FITC	553437	BD Pharmingen
Rat anti-Mouse CD45R/B220	RA3-6B2	APC	553092	BD Pharmingen
Rat anti-Mouse CD45R/B220	RA3-6B2	PE	553090	BD Pharmingen
Rat anti-Mouse CD4	RM4.5	PE-Cy7	552775	BD Pharmingen
Rat anti-Mouse CD4	GK1.5	FITC	553729	BD Pharmingen
Rat anti-Mouse CD8a	53-6.7	PE	553033	BD Pharmingen
Mouse anti-Mouse CD45.2	104	PerCP-Cy5.5	552950	BD Pharmingen
Mouse anti-Mouse CD45.1	A20	FITC	553775	BD Pharmingen

Supplementary Table 2. qPCR primer list.

Gene	Forward primer	Reverse primer
mAsxl2	CAGTCCACTGAACCCTGATATG	GGTGGCCTCTTCATCAGTAAG
mAsxl1	TCTACAGAGTCTCAGAGCCG	AGCATAACCCCAGTCCTTTTC
mPrdm16	CAGCACGGTGAAGCCATTC	GCGTGCATCCGCTTGTG
mFas	GCGATGAAGAGCATGGTTTAG	GGCTCAAGGGTTCCATGTT
mCasp3	AGATGGCTTGCCAGAAGATAC	CTGCAAAGGGACTGGATGAA
mActb	GGCTGTATTCCCCTCCATCG	CCAGTTGGTAACAATGCCATGT

Supplementary Table 3. The disease frequencies in the recipient mice.

	WT (%)	*Asxl2*$^{+/-}$ (%)	*Asxl2*$^{-/-}$ (%)
MDS	0	55.6	18.2
Leukemia	0	44.4	81.8
